# Effects of the Jump Step Kids Program on Functional Movement and Self-Report Outcomes in Children Aged 7 to 12 Years With Chronic Ankle Instability: Randomized Controlled Trial

**DOI:** 10.2196/81860

**Published:** 2025-12-19

**Authors:** Kitiyawadee Srisim, Supannikar Yingyongsaksri, Janya Chuadthong

**Affiliations:** 1 Department of Physical Therapy, School of Integrative Medicine, Mae Fah Luang University, 333 Moo 1, Thasud Subdistrict, Muang District, Chiang Rai, 57100, Thailand, 66 53916611; 2 Faculty of Physical Therapy, Mahidol University, Nakhon Pathom, Thailand; 3 Research Group on Smart Integrative Medicine and Technology Sustainability, Mae Fah Luang University, Chiang Rai, 57100, Thailand

**Keywords:** chronic ankle instability, children, pediatric rehabilitation, functional movement, ankle strength, dynamic balance, agility, motor proficiency, Jump Step Kids program, randomized controlled trial

## Abstract

**Background:**

Chronic ankle instability (CAI) is a common musculoskeletal problem in children, characterized by mechanical instability, perceived instability, and recurrent sprains. Inadequate rehabilitation can cause symptoms to persist, increase the risk of reinjury, and negatively affect long-term quality of life. Research on the rehabilitation of children with CAI concerning ankle stability, running speed, and agility has been limited. Therefore, the Jump Step Kids (JSKs) program was developed as a rhythmic, multidirectional jumping program to improve functional movement and agility in children with CAI.

**Objective:**

This study aimed to investigate the effects of the JSKs program on functional movement and self-reported outcomes in children aged 7 to 12 years with CAI.

**Methods:**

A stratified, randomized controlled trial was conducted involving 34 school-aged children with CAI. Participants were randomly allocated to either the intervention group (n=17), which underwent the JSKs program supervised by a physiotherapist, or the control group (n=17), which performed a home-based stretching program for ankle instability. Both groups participated in 30-minute sessions, 3 times per week, for 4 weeks. Outcome measurements were the heel raise test (HRT), standing long jump test, 6-meter crossover hop test, the Bruininks-Oseretsky Test of Motor Proficiency-second edition (BOT-2), and the Foot and Ankle Ability Measure questionnaire. Assessments were performed at baseline and after 4 weeks of training.

**Results:**

After 4 weeks, both groups improved in the 6-meter crossover hop test (JSKs: mean 6.60, SD 2.47 s; *P*=.02; and control: mean 6.82, SD 3.45 s; *P*=.04). The JSKs group performed better than the control group in the HRT (mean 37.88, SD 11.85 repetitions vs mean 27.65, SD 7.65 repetitions; *P*=.005), whereas the standing long jump test improved in the control group (mean 104.56, SD 21.88 cm to mean 112.05, SD 19.95 cm; *P*=.04). For BOT-2 outcomes, the 1-legged stationary hop decreased in controls (mean 35.35, SD 5.45 repetitions to mean 30.24, SD 9.21 repetitions; *P*=.01), with significant between-group differences (mean 38.18, SD 9.53 repetitions vs mean 30.24, SD 9.21 repetitions; *P*=.02). The 2-legged side hop improved in both groups (JSKs: mean 12.24, SD 4.54 repetitions to mean 25.24, SD 7.40 repetitions; control: mean 11.47, SD 3.81 repetitions to mean 26.53, SD 6.88 repetitions; *P*<.001). Self-reported outcomes, including Foot and Ankle Ability Measure-Activities of Daily Living and sports scores, did not differ significantly between groups (*P*>.05).

**Conclusions:**

The 4-week JSKs program improved functional performance in children with CAI compared with the control group in HRT and BOT-2 outcomes, although self-reported function remained unchanged. These findings provide preliminary evidence supporting the potential efficacy of the JSKs program for children with CAI. The program may serve as an addition to conventional therapy to improve physical performance in the pediatric population.

## Introduction

Chronic ankle instability (CAI) is a common musculoskeletal condition, especially among active populations, notably children [1]. CAI consists of mechanical instability (MI), recurrent sprains, and perceived instability, which can occur separately or together [2]. In children, the prevalence is notably high in those with a history of lateral ankle sprain, 23% to 71% develop instability symptoms, and 18% to 47% develop MI [1]. A recent study on school-aged children found that 36.6% of those aged 7 to 12 met CAI criteria, with MI, perceived instability, and recurrent sprains reported at 11.6%, 35.3%, and 27.3%, respectively [3].

An updated CAI model suggests that primary injury to the lateral ankle ligaments may cause sensory-perceptual and motor-behavioral impairments, which in turn impact clinical outcomes [4]. These include joint laxity, pain, reduced range of motion, decreased joint position sense, altered reflexes, and perceived instability, all of which negatively affect quality of life. In children, ankle instability has been linked to reduced functional movement, such as standing long jump performance, balance, and physical activity, compared with those without instability [5]. While perceived ankle instability influences function, self-reported outcomes, such as the Foot and Ankle Ability Measure (FAAM), have not shown noticeable differences between groups [6]. Additionally, other functional aspects—such as ankle function, stability, running speed, and agility—have not been systematically evaluated or trained in children with CAI.

Inadequate rehabilitation can extend symptoms, raise reinjury risk, and compromise long-term quality of life [4]. Therefore, effective programs are crucial. Plyometric training, which utilizes rapid stretch-shortening cycles to enhance neuromuscular control, has been demonstrated to improve balance, power, agility, and functional performance in individuals with functional ankle instability [7]. However, traditional programs may lack engagement and reduce adherence, especially in children. Recent studies using exergaming and active video games have demonstrated benefits in balance, foot and ankle function, and motivation in children with CAI, but their progression and challenge levels remain limited [6,8].

To address this gap, the Jump Step Kids (JSKs) program was created as a structured, plyometric-based intervention designed for children with CAI. Incorporating rhythm, music, and agility-based jump patterns, the program aims to improve functional outcomes while maintaining an enjoyable experience. This study examines the effects of the JSKs program on functional movement and self-reported outcomes in children aged 7 to 12 years with CAI. We hypothesized that participation would improve functional movement and foot and ankle ability, offering practical guidance for pediatric rehabilitation programs.

## Methods

### Study Design and Participants

This study was a single-blind (assessor-blind), stratified randomized controlled trial. The sample size was calculated using G*Power (v3.1.9.4; Heinrich Heine University Düsseldorf) for comparing 2 independent means, with *α*=.05 and a power of 0.80 [9], accounting for a 20% dropout rate, resulting in 34 participants (17 per group).

Thirty-four children aged 7 to 12 years with CAI from rural primary schools were recruited to participate in this study. The inclusion criteria are as follows: (1) a history of unilateral ankle sprain at least 1 year before the study [10], (2) had experienced ankle instability or *giving way* at least twice within the 6 months before the study [10], (3) Cumberland Ankle Instability Tool-Youth Thai version score ≤25 [9], (4) BMI within the normal range (5th to 85th percentile) according to the United States Centers for Disease Control and Prevention (CDC) growth chart [11], (5) no pain in the lower extremities at the time of participation, (6) ability to understand and follow instructions given by the researchers, and (7) ability to recognize and read numbers 1 to 9. The exclusion criteria were as follows: (1) acute ankle injury within 3 months before the study, (2) presence of comorbidities such as heart disease, attention deficit hyperactivity disorder, autism spectrum disorder, or asthma, (3) foot deformities, (4) history of surgery on the lower extremities, (5) history of bone fractures in the lower limbs, (6) uncontrolled health conditions including seizure disorders or uncorrected vision problems, and (7) receiving other ongoing treatments related to lower limb injuries.

### Ethical Considerations

This study was approved by the Human Research Ethics Committee of Mae Fah Luang University (EC 23146‐25, COA No. 250/2023) and conducted in accordance with the Declaration of Helsinki. Before participation, written informed consent was obtained from the legal guardians, and an assent form for the children included in the study guaranteed the protection of participants’ rights and safety. All research activities were conducted in accordance with ethical standards, including strict adherence to privacy and confidentiality principles, to protect the data and identities of participants. Participants received compensation of 100 Thai Baht per visit to cover time and travel expenses, with no coercive intent.

### Outcome Measurements

#### Overview

The 34 eligible participants were randomly stratified by age and gender and assigned to 2 groups: 17 participants received the JSKs program, and 17 participants received a home-based stretching program. Functional movement and self-reported outcomes were assessed before and after the 4-week training period. All assessments were conducted by a single trained assessor who was blinded to the group assignment. The assessments included the following tests.

#### Heel Raise Test

The heel raise test (HRT) was used to assess calf muscle strength and endurance [12]. Before testing, participants performed their highest possible heel raise to establish a reference height. They were then instructed to perform as many single-leg heel raises as possible within 30 seconds. The number of valid repetitions—completed without allowing the non-testing foot to touch the ground or losing balance—was recorded for analysis [13].

#### Standing Long Jump Test

The standing long jump test (SLJT), a strength subtest of the Bruininks-Oseretsky Test of Motor Proficiency, Second Edition (BOT-2), was used to assess lower extremity muscle strength [14]. Participants stood behind a starting line with their feet shoulder-width apart and performed a forward jump as far as possible. The distance from the starting line to the nearest heel upon landing was measured in centimeters. Each participant completed 1 trial, with a second trial permitted only if they stumbled or fell during the first attempt.

#### Six-Meter Crossover Hop Test

The 6-meter crossover hop test (6mCHT) is a dynamic test that assesses ankle function and stability. Participants stood on their injured leg, arms free, and jumped as fast as they could in a diagonal pattern across 2 parallel lines spaced 15 cm apart for 6 meters. The test was repeated if the untested foot did not contact the ground, lost balance, or crossed the tape line incorrectly. The assessor recorded the time taken in seconds. The test was performed 3 times, and the average time was analyzed [15].

#### Shuttle Run

The shuttle run, a subtest of the BOT-2, was used to assess speed and agility in children [14]. Participants stood behind the starting line and sprinted as fast as possible to retrieve blocks placed 15.24 meters away, then returned to the starting line. One trial was performed, with a second trial administered only if the participant tripped or failed to retrieve the blocks. The time in seconds was used for analysis [14].

#### Stepping Sideways Over a Balance Beam

This is a running speed and agility subtest of the BOT-2. Participants stood beside the beam with feet together and hands on hips, then stepped laterally across it, alternating feet continuously for 15 seconds. A second trial was allowed only if they tripped or fell. The number of correct steps was recorded for analysis [14].

#### One-Legged Stationary Hop

This is another running speed and agility subtest of the BOT-2. The participants stood on the injured leg, hands on the hips, then hopped up and down on the injured leg for 15 seconds. A second attempt was performed only if the subject tripped or fell during the first attempt. The number of correct attempts for statistical analysis [14].

#### One-Legged Side Hop

This is also a running speed and agility subtest of the BOT-2. Participants stood on the injured leg behind a line, hands on hips, and hopped vertically on the injured leg across the line to the left and right continuously for 15 seconds. A second test was performed if the participant tripped or fell during the test. The number of successful jumps was used for analysis [14].

#### Two-Legged Side Hop

This is a running speed and agility subtest of the BOT-2. Participants stood feet together parallel to a line with hands on hips, hopped both feet sideways across the line simultaneously for 15 seconds. A second trial is performed if they trip or fall. The number of correct hops was recorded [14].

#### FAAM-Thai Version

The FAAM is a 5-point scale self-reported questionnaire used for foot and ankle conditions [16]. It comprises 2 subscales including the 21 Activities of Daily Living (ADL) and 8 Sports scale, with a total score of 84 (ADL) and 32 (Sports), then converted into percentages (100%=full function; 0%=complete disability). Items not completed for reasons unrelated to foot or ankle conditions were marked as “not applicable or N/A” and excluded from the final score [17]. Participants reported their function over the past week. To ensure reliability, the FAAM questionnaire was conducted by a single trained researcher who interviewed each participant throughout the study period.

### Intervention Programs

Participants were randomly assigned to either the intervention or control group, with sessions lasting 30 minutes, 3 times a week, over 4 weeks.

#### The JSKs Program

The JSKs program is supervised by a licensed physical therapist with over 15 years of experience working with children. All participants were instructed to perform the intervention under the guidance of a physical therapist. Each session includes 3 parts: a 5-minute warm-up, a 20-minute core exercise period, and a 5-minute cool-down. All sessions are carried out using a 3×3 agility training grid.

#### Warm-Up and Cool-Down

**Figure 1.**
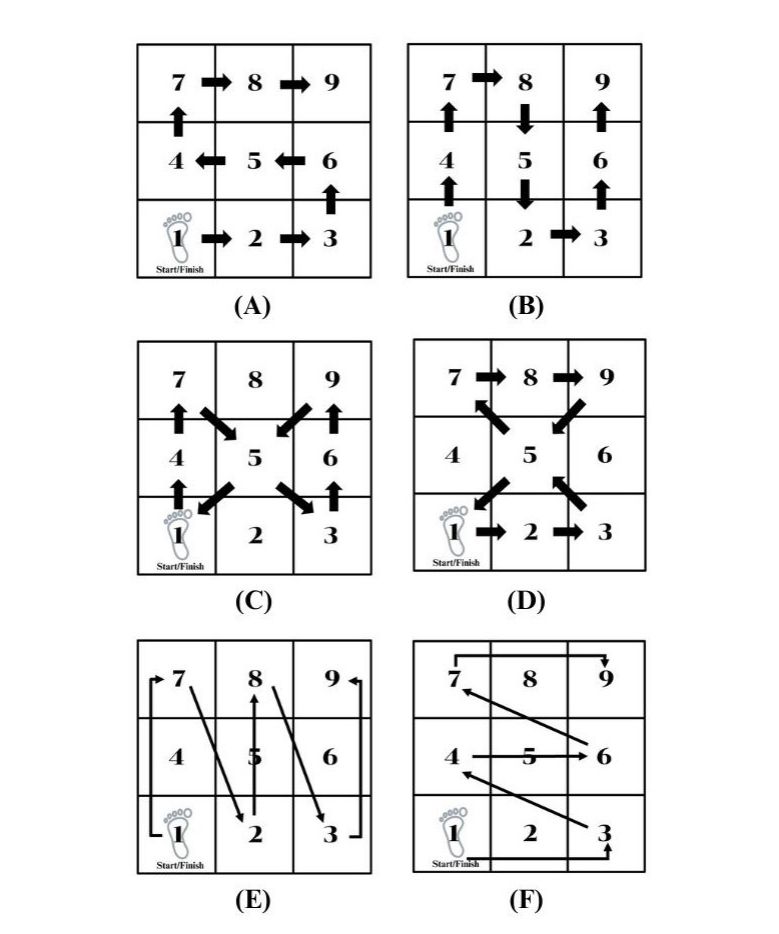
Jump Step Kids program exercises: (A) 1-leg lateral side hopping; (B) 1-leg forward-backward hopping; (C) 1-leg figure-of-eight hopping (vertical pattern); (D) 1-leg figure-of-eight hopping (horizontal pattern); (E) 1-leg long jump (N-Shape pattern); and (F) 1-leg long jump (Z-shape pattern).

Participants stood on the center mat (square 5 of a 3×3 agility grid) and performed dynamic stretching by stepping 1 leg as far as possible in 8 directions adapted from the Star Excursion Balance Test [18]. The order for stepping the left leg started from numbers 9 to 6, and the right leg started from numbers 7 to 4 (Figure 1). Then, they were instructed to practice a single-leg stand with eyes closed for 30 seconds each side.

#### Main Exercise

##### Overview

Participants completed a 20-minute JSKs program, consisting of 4 progressive tasks, each lasting 5 minutes (Figure 1). Exercises were presented in sequence, from easy to difficult, moving from Exercise 1 to Exercise 4. Surface conditions changed from a hard surface during weeks 1 to 2 to a foam surface in weeks 3 to 4. All movements were demonstrated via video and practiced on both sides of the body.

##### Exercise 1: 1-Leg Lateral Side Hopping

Participants stood on 1 leg in square 1 with their hands on their hips and performed single-leg lateral hops across the grid in this sequence: 1 → 9 (Figure 1A). When they reached square 9, they balanced statically for 5 seconds, then reversed the path back to square 1, followed by another 5-second pause. The exact sequence was then repeated on the other leg. Each leg completed 3 sets.

##### Exercise 2: 1-Leg Forward-Backward Hopping

Participants stood on the supporting leg in square 7 with their hands on their hips and performed single-leg forward and backward hops along the sequence: 1 → 9 (Figure 1B). Upon reaching square 9, they maintained balance for 5 seconds, then returned along the same path to square 1, followed by another 5-second pause. The sequence was repeated on the opposite leg. Each leg completed 3 sets.

##### Exercise 3: 1-Leg Figure-of-Eight Hopping

Participants stood on the supporting leg in square 1 with their hands on their hips and performed single-leg hops following a vertical figure-of-eight pattern: 1 → 1 (Figure 1C, vertical pattern). After completing the sequence, they paused for 5 seconds before repeating the task on the opposite leg. Each leg completed 3 sets. Then, participants stood on the supporting leg in square 1 with their hands on their hips and performed single-leg hops following a horizontal figure-of-eight pattern: 1 → 1 (Figure 1D, horizontal pattern). After finishing the sequence, they paused for 5 seconds and repeated the task on the opposite leg. Each leg completed 3 sets.

##### Exercise 4: 1-Leg Long Jump

Participants stood on the supporting leg in square 1 with their hands on their hips and performed single-leg long jumps laterally, following an N-shaped sequence: 1 → 9. Upon reaching square 9, they paused for 5 seconds before returning along the same path in reverse order (Figure 1E, N-Shape Pattern). A second 5-second pause was observed upon return. The task was then repeated on the opposite leg. Each leg completed one full N-pattern in both directions. After that, participants stood on the supporting leg in square 1 with their hands on their hips and executed single-leg long jumps along a Z-shaped sequence: 1 → 9. After reaching square 9, they paused for 5 seconds before retracing the path in reverse order (Figure 1F, Z-Shape Pattern). A final 5-second pause was observed at square 1. The sequence was repeated on the opposite leg. Each leg completed one full Z-pattern in both directions.

### Control Group Program

Participants were asked to follow a home-based stretching program outlined in the brochure. This program is adapted from a general exercise used in their school. It included 6 exercises designed to actively stretch the hip, knee, and ankle muscles in sitting and standing positions. Each exercise was held for 15 seconds and repeated 10 times, with 3 sets per leg. Each session lasted approximately 30 minutes, 3 times a week, for a period of 4 weeks. A researcher contacted the participants to remind them and their parents to follow the stretching protocol. Additionally, all participants recorded their stretching sessions in a child logbook throughout the 4 weeks.

### Statistical Analysis

The data were analyzed using SPSS (version 20.0; IBM Corp). Descriptive statistics are shown as mean (SD) for continuous variables and frequency (percentage) for categorical variables. The normality of continuous data was checked with the Shapiro-Wilk test. Between-group differences were tested using univariate analysis of covariance (ANCOVA) with Bonferroni post hoc corrections. Within-group differences were examined using two-tailed paired sample *t* tests. A *P*<.05 was considered statistically significant.

## Results

**Figure 2.**
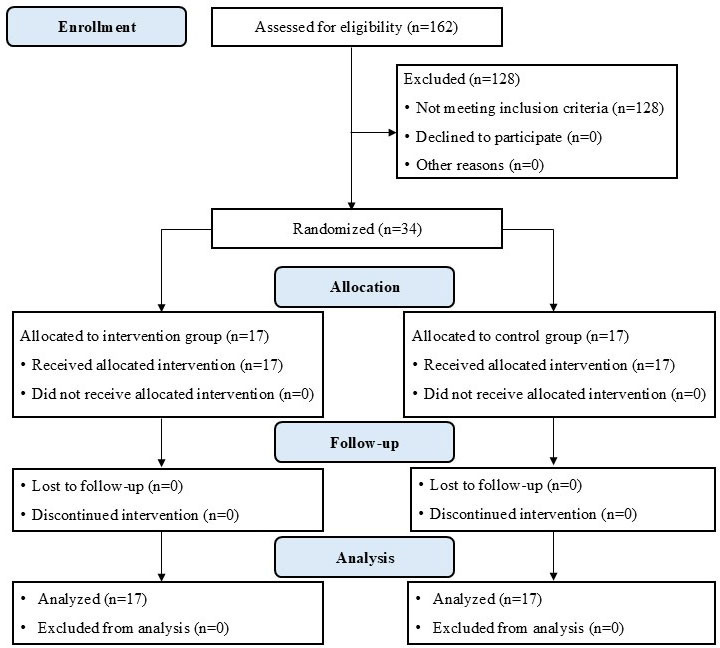
The CONSORT (Consolidated Standards of Reporting Trials) flow diagram of the study participant recruitment, allocation, follow-up, and analysis.

A total of 34 children with CAI were analyzed. Participant enrollment, allocation, follow-up, and analysis are presented in the CONSORT (Consolidated Standards of Reporting Trials) flow diagram (Figure 2). In the JSKs program group, all participants completed all 12 training sessions as scheduled over a 4-week period. Similarly, in the control group, all participants attended every scheduled session. Descriptive statistics were used to describe the baseline characteristics of participants, including sex, age, height, weight, side of CAI, and Cumberland Ankle Instability Tool-Youth Thai version scores. No significant differences were found between the JSKs program and control groups at baseline (*P*>.05; Table 1).

**Table 1.** Baseline characteristics of the participants (N=34).

Variable	JSKs^a^ program group (n=17)	Control group (n=17)	*P* value
Age (years), mean (SD)	9.39 (2.05)	9.09 (1.72)	.64
Weight (kg), mean (SD)	31.06 (10.85)	32.06 (8.93)	.77
Height (cm), mean (SD)	134.62 (14.35)	131.68 (9.02)	.48
CAITY-T^b^ score, mean (SD)	18.65 (4.37)	16.41 (4.72)	.16
Sex, n (%)			.49
Female	9 (53)	7 (41)	
Male	8 (47)	10 (59)	
Side of chronic ankle instability, n (%)			>.99
Right	10 (59)	10 (59)	
Left	7 (41)	7 (41)	

a JSK: Jump Step Kids.

b CAITY-T: Cumberland Ankle Instability Tool-Youth Thai version.

Foot and ankle function outcomes were assessed within and between groups at baseline and after 4 weeks of training. Within the JSKs program group, significant improvements were observed in the 6mCHT after week 4 (*P*=.02). Similarly, the 2-legged side hop test of BOT-2 showed a significantly increased score (*P*<.001). No significant within-group changes were observed in the HRT, SLJT, shuttle run, sideways stepping over a balance beam, or FAAM scores in the JSKs group (*P*>.05).

In the control group, there was a significant decrease in the 6mCHT at week 4 (*P*=.04), and the 1-legged stationary hop test also decreased significantly (*P*=.01). Additionally, there was a significant improvement in the SLJT (*P*=.04), and the 2-legged side hop test of BOT2 increased substantially in the control group (*P*<.001). Other outcomes did not show significant changes over the 4 weeks (Table 2).

Between-group comparisons using ANCOVA adjusted for baseline values revealed significant differences for the HRT (*F*_1,32_=4.39; *P*=.005; ηp²=0.12) and the 1-legged stationary hop test of BOT-2 (*F*_1,32_=6.17; *P*=.02; ηp²=0.20), with the JSKs program group showing greater improvements than the control group at week 4. Other outcomes did not change over the 4-week training period (Table 2).

**Table 2.** Comparison within and between-group analysis of functional movement and self-reported outcomes between Jump Step Kids (JSKs) program and control groups.

Variables	JSKs^a^ program group (n=17)			Control group (n=17)			Effect size (Cohen *d*)	Between groups *P* value	95% CI
	Baseline, mean (SD)	Week 4, mean (SD)	*P* value	Baseline, mean (SD)	Week 4, mean (SD)	*P* value			
Heel raise test (repetition)	36.47 (9.01)	37.88 (11.85)	.60	30.35 (6.95)	27.65 (7.65)	.23	−1.026	.04^b^	3.26 to 17.20
Standing long jump test (cm)	107.85 (21.95)	113.23 (26.44)	.33	104.56 (21.88)	112.05 (19.95)	.04^c^	0.073	.26	−6.24 to 25.73
Six-meter crossover hop test (second)	8.43 (4.07)	6.60 (2.47)	.02^c^	9.54 (6.39)	6.82 (3.45)	.04^c^	0.073	.28	−2.41 to 1.98
Shuttle run (second)	9.27 (1.25)	8.96 (1.10)	.11	9.13 (2.42)	9.06 (1.16)	.88	0.09	.64	−0.89 to 0.69
Stepping sideways over a balance beam (repetition)	29.65 (6.59)	30.47 (5.20)	.58	28.00 (7.37)	30.65 (5.45)	.12	0.034	.17	−3.90 to 3.55
One-legged stationary hop (repetition)	35.88 (7.31)	38.18 (9.53)	.14	35.35 (5.45)	30.24 (9.21)	.01^c^	−0.847	.02^b^	1.39 to 14.49
One-legged side hop (repetition)	21.94 (6.30)	23.88 (8.72)	.28	16.29 (5.03)	20.56 (9.00)	.12	−0.375	.81	−2.97 to 9.61
Two-legged side hop (repetition)	12.24 (4.54)	25.24 (7.40)	<.001^c^	11.47 (3.81)	26.53 (6.88)	<.001^c^	0.181	.62	−6.28 to 3.69
FAAM-ADL^d^ (%)	85.97 (10.06)	86.62 (13.02)	.81	87.69 (5.93)	86.43 (6.09)	.36	−0.019	.59	−6.91 to 7.29
FAAM-Sport^e^ (%)	85.59 (14.38)	84.30 (15.91)	.53	86.00 (10.69)	89.35 (6.24)	.52	0.418	.43	−13.49 to 3.39

a JSKs: Jump Step Kids.

b Between-group comparisons were analyzed using univariate ANCOVA with Bonferroni post hoc adjustments.

c Within-group comparisons were analyzed using paired *t* tests.

d FAAM-ADL: Foot and Ankle Ability Measure-Activities of Daily Living.

e FAAM-Sport: Foot and Ankle Ability Measure-Sport.

## Discussion

### Principal Findings

This study is the first to evaluate a program designed to improve functional movement and agility in children with CAI. The purpose of this study was to investigate the effect of the JSKs program on functional movement and self-reported outcomes in children with CAI. After 4 weeks, the JSKs program significantly enhanced the 6mCHT (*P*=.02) and 2-legged side hop (*P*<.001), with improvements in the HRT (*P*=.04) and 1-legged stationary hop (*P*=.02) observed between groups. The control group improved in SLJT (*P*=.04) and 2-legged side hop (*P*<.001) but declined in HRT and 1-legged stationary hop (*P*=.01). No significant differences were found for FAAM-ADL or Sport (*P*>.05).

The effects of the intervention on functional muscle strength were evaluated using the HRT and the SLJT. The HRT was used to assess both the strength and endurance of the ankle plantarflexor and dorsiflexor muscle groups [13,19]. After 4 weeks of training, the HRT performance remained stable in the JSKs group, while the control group showed a decline in this performance. The absence of improvement in ankle strength within the JSKs group may be attributed to the nature of the training program, which primarily consists of multidirectional jumping tasks. These exercises emphasize the development of explosive power, neuromuscular responsiveness, and dynamic muscle strengthening, rather than isolated static muscle strengthening or slow, fully controlled concentric contractions—precisely the type of muscular demand assessed by the HRT [20,21]. In addition, although jumping exercises use body weight as resistance, they focus on building momentum rather than maintaining a constant muscle load. In contrast, the HRT involves continuous, controlled resistance, thereby offering a more accurate measure of calf muscle endurance and strength [21,22]. Consequently, it is likely that the JSKs program did not provide sufficient training stimulus to induce measurable gains in ankle muscle strength. Nevertheless, this study demonstrates that the JSKs program was effective in maintaining ankle muscle strength in children with CAI when compared to the control group.

In contrast, the SLJT is widely used to assess overall lower extremity muscle strength and explosive power and requires coordinated force generation from both legs [14,23]. In this study, only the control group revealed a statistically significant improvement in jump distance (from 104.56 cm to 112.05 cm). This improvement may be attributed to increased muscle flexibility and joint range of motion resulting from regular stretching, which can increase muscle compliance and optimize force production during jumping [24]. In contrast, the JSKs group demonstrated a nonsignificant increase in jump distance (from 107.85 cm to 113.23 cm). The JSKs program emphasizes multidirectional jumping activities, focusing on balance, speed, and agility rather than maximal jump distance. Consistent with Legnani et al [25], who demonstrated that side hop tests could be more accurate than vertical jump tests for detecting functional impairment in patients suffering from CAI, our findings may explain the lack of significant improvement in SLJT performance in this group. Moreover, the bilateral nature of the SLJT may conceal unilateral impairments, supporting prior recommendations for single-leg assessments using force plates [26,27].

The 6mCHT assessed dynamic balance capability and ankle stability [15]. The findings of this study indicated a significant improvement in both groups after 4 weeks. The JSKs group demonstrated improvements through program components that promoted multidirectional jumping, dynamic balance, and lower extremity coordination. This training has likely enhanced ankle stability, body position awareness, and motor control, which are essential for effective performance in crossover hopping tasks. The findings align with a prior study by Myer et al [28], who indicated that youth athletes exhibited enhanced dynamic stability and hop performance after engaging in plyometric and neuromuscular training. Moreover, the control group also showed significant improvements over 4 weeks of training. These changes may result from natural developmental progress, increased awareness of physical activity during the study period, or learning effects linked to repeated test exposure. Gribble et al [29] observed that performance increased on repeated hop tests without specific training. This improved performance may be due to task familiarity and motor learning. However, the magnitude of improvement was greater in the JSKs group, reinforcing the efficacy of a structured, progressive training program. The JSKs protocol, which included exercises progressing from stable to unstable surfaces, is consistent with the approach used in the previous study [30]. Eils and Rosenbaum [30] found improved hop performance after a balance-focused intervention in individuals with CAI. Therefore, this study suggested that multidirectional jumping activity in the JSKs program could be beneficial for ankle movement to maintain dynamic balance control for children with CAI.

For the agility test, it was assessed using the BOT-2 subtests of running speed and agility. Agility is an important characteristic of motor development, which is crucial for maintaining and controlling body posture when changing direction. The results of this study indicated that both the JSKs program and a self-stretching program increased the number of jumps in 2-legged side hop test after 4 weeks of training. These findings support the role of agility as an important aspect of motor development, which is closely related to physical coordination, speed, and balance skills [31]. In the experimental group receiving the JSKs program, the children practiced alternating forward-backward and side-to-side hops, stimulating the central nervous system and the proprioceptive system through postural control and rapid direction changes in movement. These findings align with previous studies showing that hop-based training enhances ankle stability, functional performance, and postural control in individuals with CAI [32,33]. In addition, Hale et al [34] aimed to examine the effects of a 4-week rehabilitation program for CAI on postural control and lower extremity function. This comprehensive rehabilitation program consisted of range of motion exercises, strength exercises, neuromuscular control, and functional tasks. Their findings showed that rehabilitation effectively improves postural control and functional limitations in individuals with CAI. In addition, the results showed a significant decrease in the 1-legged stationary hop test in control groups when compared with the JSKs group. This finding suggests that the JSKs program is more effective in enhancing single-limb control, unilateral coordination, and maintaining strength over time. These findings are supported by previous studies which demonstrated that hopping, balance, and proprioceptive training can improve motor control and stability in children with CAI [26,28]. Thus, this finding suggests that the JSKs program has an impact on the improvement or maintenance of performance in children with CAI. Although the 2-legged hop test does not fully replicate real-world situations, it provides a safe and specific test of agility for children, supporting the development of coordination, balance, and spatial awareness. These skills are essential for activities requiring speed, such as running, avoiding obstacles, playing sports, and participating in physical education, thereby enhancing safety in daily life.

The FAAM was used to assess physical functioning in individuals with foot and ankle disorders [18]. Self-reported questionnaires may be influenced by children’s mood, confidence, or understanding, as they reflect perceived rather than actual physical changes. This study revealed no statistically significant differences between the intervention and control groups after 4 weeks of training. This finding was similar to Kim et al [35], who also reported no difference between a jumping training and control group even though the training period was 6 weeks in adults with CAI. In contrast, Kim et al [36] and Ardakani et al [37] reported both FAAM-ADL and FAAM-Sport improved in hop-stabilization and balance training between pre- and posttraining for 6 to 8 weeks. The 4-week training period in this study may be too short to produce significant changes in self-perceived ability, especially in children with CAI [23]. The authors of the present study further suggest that longer interventions may be necessary.

### Limitations

This study only investigated the short-term effects of the JSKs program on functional movement and self-reported outcomes related to the foot and ankle in children with CAI. Therefore, future research should include ongoing follow-up to assess the sustainability of the training effects over time. Additionally, this study did not evaluate factors associated with ankle function and movement control that could help elucidate the underlying mechanisms behind the observed outcomes. Future studies should incorporate additional assessments, such as measurements of muscle strength, to gain a more comprehensive understanding of the training effects. Furthermore, the FAAM used in this study has not been validated for pediatric populations, which may limit the interpretability of the self-reported functional outcomes. Therefore, future studies should consider using or developing validated outcome measures for children to ensure more accurate assessment of ankle function.

### Conclusions

After 4 weeks of training, the JSKs program showed greater improvement in functional movement than the home-based stretching program in children aged 7 to 12 years with CAI, as measured by HRT and the 1-legged stationary hop test. Additionally, both training programs enhance performance in the 6mCHT and 2-legged side hop. However, the self-reported outcomes (FAAM-ADL and FAAM-Sport subscales) did not change between groups. For clinical applications, the JSKs program offers a practical, accessible, and time-efficient plyometric training protocol that can be easily integrated into the rehabilitation of children with CAI. Clinicians should consider incorporating this program alongside conventional treatment to promote ankle stability and potentially reduce the risk of recurrent ankle sprains.

## Supplementary material

10.2196/81860 Checklist 1 CONSORT-eHEALTH checklist (V 1.6.1).

## Abbreviations

6mCHT: 6-meter crossover hop test
ADL: activity of daily living
BOT-2: Bruininks-Oseretsky Test of Motor Proficiency, Second Edition
CAI: chronic ankle instability
CDC: Centers for Disease Control and Prevention
FAAM: Foot and Ankle Ability Measure
HRT: heel raise test
JSKs: Jump Step Kids
MI: mechanical instability
SLJT: standing long jump test

## References

[1] Mandarakas M, Pourkazemi F, Sman A, Burns J, Hiller CE (2014). Systematic review of chronic ankle instability in children. J Foot Ankle Res.

[2] Hiller CE, Kilbreath SL, Refshauge KM (2011). Chronic ankle instability: evolution of the model. J Athl Train.

[3] Lekskulchai R, Kadli S (2020). Prevalence and factors associated with chronic ankle instability among children aged 7 to 12 years. J Assoc Med Sci.

[4] Hertel J, Corbett RO (2019). An updated model of chronic ankle instability. J Athl Train.

[5] Suphasubtrakul T, Lekskulchai R, Jalayondeja C (2024). Balance, strength and physical activity after ankle sprain: comparison between children with chronic ankle instability and copers. Phys Ther Sport.

[6] Chuadthong J, Lekskulchai R, Hiller C, Ajjimaporn A (2023). A home-based exercise program with active video games for balance, motor proficiency, foot and ankle ability, and intrinsic motivation in children with chronic ankle instability: feasibility randomized controlled trial. JMIR Serious Games.

[7] Surakhamhaeng A, Bovonsunthonchai S, Vachalathiti R (2020). Effects of balance and plyometric training on balance control among individuals with functional ankle instability. Physiother Quart.

[8] Fitzgerald D, Trakarnratanakul N, Smyth B, Caulfield B (2010). Effects of a wobble board-based therapeutic exergaming system for balance training on dynamic postural stability and intrinsic motivation levels. J Orthop Sports Phys Ther.

[9] Yingyongsaksri S, Hiller CE, Tharawadeepimuk K, Nanbancha A (2023). Reliability and validation of the Thai version of the Cumberland Ankle Instability Tool (CAIT-THA). Disabil Rehabil.

[10] Gribble PA, Delahunt E, Bleakley C (2013). Selection criteria for patients with chronic ankle instability in controlled research: a position statement of the International Ankle Consortium. J Orthop Sports Phys Ther.

[11] Hales CM, Freedman DS, Akinbami L, Wei R, Ogden CL (2022). Evaluation of alternative body mass index (BMI) metrics to monitor weight status in children and adolescents with extremely high BMI using CDC BMI-for-age growth charts. Vital Health Stat 1.

[12] Sman AD, Hiller CE, Imer A, Ocsing A, Burns J, Refshauge KM (2014). Design and reliability of a novel heel rise test measuring device for plantarflexion endurance. Biomed Res Int.

[13] Yocum A, McCoy SW, Bjornson KF, Mullens P, Burton GN (2010). Reliability and validity of the standing heel-rise test. Phys Occup Ther Pediatr.

[14] Bruininks RH, Bruininks BD (2005). Bruininks-Oseretsky Test of Motor Proficiency.

[15] Yalfani A, Gandomi F, Kohboomi M (2017). The effect of G-max and G-med muscle fatigue on functional performance and balance in athletes with and without chronic ankle instability. Asian J Sports Med.

[16] Arunakul M, Arunakul P, Suesiritumrong C, Angthong C, Chernchujit B (2015). Validity and reliability of Thai version of the foot and ankle ability measure (FAAM) subjective form. J Med Assoc Thai.

[17] Martin RL, Irrgang JJ, Burdett RG, Conti SF, Van Swearingen JM (2005). Evidence of validity for the foot and ankle ability measure (FAAM). Foot Ankle Int.

[18] Gribble PA, Hertel J, Plisky P (2012). Using the Star Excursion Balance Test to assess dynamic postural-control deficits and outcomes in lower extremity injury: a literature and systematic review. J Athl Train.

[19] Bohannon RW (2022). The heel-raise test for ankle plantarflexor strength: a scoping review and meta-analysis of studies providing norms. J Phys Ther Sci.

[20] Kadlubowski B, Keiner M, Wirth K, Csapo R (2025). Effects of traditional strength vs. combined strength and plyometric training on sprint, jump, and maximum strength performance in elite youth soccer players-a 6-month controlled trial. J Strength Cond Res.

[21] Ramírez-delaCruz M, Bravo-Sánchez A, Esteban-García P, Jiménez F, Abián-Vicén J (2022). Effects of plyometric training on lower body muscle architecture, tendon structure, stiffness and physical performance: a systematic review and meta-analysis. Sports Med Open.

[22] Markovic G, Mikulic P (2010). Neuro-musculoskeletal and performance adaptations to lower-extremity plyometric training. Sports Med.

[23] Luan L, Zhu M, Adams R, Witchalls J, Pranata A, Han J (2023). Effects of acupuncture or similar needling therapy on pain, proprioception, balance, and self-reported function in individuals with chronic ankle instability: a systematic review and meta-analysis. Complement Ther Med.

[24] Behm DG, Chaouachi A (2011). A review of the acute effects of static and dynamic stretching on performance. Eur J Appl Physiol.

[25] Legnani C, Saladini M, Faraldi M, Peretti GM, Ventura A (2025). A battery of jump tests helps discriminating between subjects with and without chronic ankle instability. Sports (Basel).

[26] Hu X, Feng T, Li P, Liao J, Wang L (2024). Bilateral sensorimotor impairments in individuals with unilateral chronic ankle instability: a systematic review and meta-analysis. Sports Med Open.

[27] Ross SE, Guskiewicz KM, Gross MT, Yu B (2009). Balance measures for discriminating between functionally unstable and stable ankles. Med Sci Sports Exerc.

[28] Myer GD, Ford KR, Brent JL, Hewett TE (2006). The effects of plyometric vs. dynamic stabilization and balance training on power, balance, and landing force in female athletes. J Strength Cond Res.

[29] Gribble PA, Hertel J, Denegar CR, Buckley WE (2004). The effects of fatigue and chronic ankle instability on dynamic postural control. J Athl Train.

[30] Eils E, Rosenbaum D (2001). A multi-station proprioceptive exercise program in patients with ankle instability. Med Sci Sports Exerc.

[31] João PV, Simões I, Alves L, Santos L, Pereira A, Mota MP (2014). Physical activity with agility motor development for children ages 6–10. Sci Sports.

[32] Anguish B, Sandrey MA (2018). Two 4-week balance-training programs for chronic ankle instability. J Athl Train.

[33] Park HS, Oh JK, Hong YI, Kim JY, Yoon JH (2025). Effect of hop-stabilization training on ankle instability and function of adolescent female basketball players with chronic ankle instability: a double-blind, prospective, cluster-randomized controlled trial. J Clin Med.

[34] Hale SA, Hertel J, Olmsted-Kramer LC (2007). The effect of a 4-week comprehensive rehabilitation program on postural control and lower extremity function in individuals with chronic ankle instability. J Orthop Sports Phys Ther.

[35] Kim KM, Estudillo-Martínez MD, Castellote-Caballero Y, Estepa-Gallego A, Cruz-Díaz D (2021). Short-term effects of balance training with stroboscopic vision for patients with chronic ankle instability: a single-blinded randomized controlled trial. Int J Environ Res Public Health.

[36] Kim KM, Estepa-Gallego A, Estudillo-Martínez MD, Castellote-Caballero Y, Cruz-Díaz D (2022). Comparative effects of neuromuscular- and strength-training protocols on pathomechanical, sensory-perceptual, and motor-behavioral impairments in patients with chronic ankle instability: randomized controlled trial. Healthcare (Basel).

[37] Ardakani MK, Wikstrom EA, Minoonejad H, Rajabi R, Sharifnezhad A (2019). Hop-stabilization training and landing biomechanics in athletes with chronic ankle instability: a randomized controlled trial. J Athl Train.

